# FACTORS THAT INFLUENCE LIFE SATISFACTION IN AL RESIDENTS

**DOI:** 10.1093/geroni/igz038.881

**Published:** 2019-11-08

**Authors:** Sarah Holmes

**Affiliations:** University of Maryland, Baltimore, Maryland, United States

## Abstract

Life satisfaction is a multidimensional concept that addresses a personal judgment of quality from the resident’s perspective. Components of life satisfaction include satisfaction related to health, the physical environment, relationships and activities. The purpose of this study was to test if there was a relationship between demographic factors, pain, falls, and use of psychotropics with life satisfaction. The sample included the first two cohorts from the FFC-AL-EIT study including 508 residents from 54 settings across Maryland, Pennsylvania, and Massachusetts. The majority of the participants were female (70%), white (97%) and the mean age was 87.72 (SD=7.47) . Based on a stepwise linear regression analysis there was a significant association between pain (r=-.20, p=.003) and psychotropic use (r=-.19, p=.003) and the model explained 11% of the variance in life satisfaction. Ongoing research is needed to consider the impact of the environment and staff-resident interactions on life satisfaction.

